# Selected-Wavelength Illumination for Enhanced Hydrogen and Poly-β-hydroxybutyrate Production from Second Cheese Whey by *Rhodopseudomonas palustris*

**DOI:** 10.3390/microorganisms14010032

**Published:** 2025-12-22

**Authors:** Luca Bernabò, Giulia Daly, Viola Galli, Simona Guerrini, Carlo Viti, Lisa Granchi, Alessandra Adessi

**Affiliations:** 1 Department of Agriculture, Food, Environment and Forestry (DAGRI), University of Florence, Piazzale delle Cascine, 18, 50144 Florence, Italy; luca.bernabo@unifi.it (L.B.); giulia.daly@unifi.it (G.D.); viola@foodmicroteam.it (V.G.); simona.guerrini@unifi.it (S.G.); carlo.viti@unifi.it (C.V.); lisa.granchi@unifi.it (L.G.); 2 FoodMicroTeam s.r.l., Academic Spin-Off of University of Florence, Via Santo Spirito 14, 50125 Florence, Italy

**Keywords:** lactic acid bacteria, dark fermentation, photofermentation, poly-β-hydroxybutyrate, light quality, energy recovery

## Abstract

Second cheese whey (SCW), a major by-product of ricotta cheese production, poses significant environmental challenges due to its high organic load. Biohydrogen (bio-H_2_) and poly-β-hydroxybutyrate (PHB) production offer a sustainable reuse of SCW, that provides ideal nutrients for microbial growth. This study aimed to convert SCW into Bio-H_2_ and PHB using a 5-liter tubular bioreactor in a sequential lactic fermentation and photofermentation system. Two lighting conditions were tested: white LED (WL) and selected LED (SL). Optimal results were achieved with a co-inoculum of *Lactococcus lactis* MK L84 and *Lacticaseibacillus paracasei* MK L49 at pH 4.5–5.5, followed by photofermentation with *Rhodopseudomonas palustris* 42OL under SL condition. The process yielded an average of 0.47 L of H_2_ per liter of substrate and 1.66% wPHB/wCDW. This approach successfully transformed dairy waste into high-value products, promoting circular economy principles.

## 1. Introduction

Currently, most energy comes from non-renewable fossil fuels, driving climate change [1]. The EU targets a 90% reduction in greenhouse gas emissions by 2040 and climate neutrality by 2050 [2]. Hydrogen (H_2_), with zero emissions and high energy density, is a promising alternative to fossil fuels [3]. H_2_ can find applications across sectors like transportation, electricity, and industry [4]. However, fossil-fuel-based H_2_ production releases ~830 million tons of CO_2_ annually [5].

In this scenario, microbiological H_2_ production (bio-H_2_) using agro-industrial waste offers a sustainable alternative, by producing H_2_ independently of fossil fuels and converting waste into valuable resources, in line with circular economy principles [6,7]. Bio-H_2_ can be produced via bio-photolysis, dark fermentation, and photofermentation using microorganisms such as microalgae, non-photosynthetic bacteria, and photosynthetic bacteria (PSB) [8].

Integrating dark fermentation and photofermentation optimizes bio-H_2_ production, showing potential for future industrial applications [9,10]. Dark fermentation, driven by anaerobic bacteria, generates bio-H_2_, organic acids, volatile fatty acids (VFAs), and CO_2_ without light [10]. The organic acids serve as substrates for photofermentation by purple non-sulfur bacteria (PNSB), which generate bio-H_2_ via nitrogenase enzyme under nitrogen-limited conditions [11,12]. During photofermentation, PNSB grow photoheterotrophically under anaerobic conditions, using organic acids as carbon and electron sources while harnessing light as their energy source [12].

Agro-industrial wastewater is often rich in ammoniacal nitrogen, which inhibits nitrogenase activity and thus hampers bio-H_2_ production [13]. Substrate dilution is a common strategy to mitigate this issue, although this approach may increase substrate volumes and process costs [14,15].

*Rhodopseudomonas palustris* is a highly versatile PNSB for bio-H_2_ production, as it efficiently utilizes various organic substrates, including industrial waste [16,17]. Its versatility is also linked to three nitrogenase isoforms (molybdenum, vanadium, and iron), each varying based on the metal in the enzyme’s active site, allowing for bio-H_2_ production under different environmental conditions [18,19].

PNSB can produce and accumulate poly-β-hydroxybutyrate (PHB), a biodegradable polymer from the polyhydroxyalkanoates (PHA) family, suitable for bioplastics production [9]. PHB acts as an energy reserve and stress protector, synthesized under high carbon-nitrogen (C/N) ratios and nutrient-limited conditions (e.g., sulfate, phosphate, magnesium) [20]. While PHB biosynthesis competes with bio-H_2_ production as a reductive pathway, both can be simultaneously generated [21].

Dairy production plays a crucial role in human nutrition but generates millions of tons of waste per year, including cheese whey (CW) and second cheese whey (SCW), both with high environmental impact if not correctly managed [22,23,24]. SCW, produced by heating CW during ricotta cheese production, is rich in lactose (35–50 g L^−1^) and has high COD (50 g L^−1^) and BOD (80 g L^−1^) levels [24]. In Italy, 15% of CW is used to obtain ricotta cheese, producing about 1 million tons of SCW per year. Though often reused as animal feed, SCW contains valuable compounds like proteins, peptides, and lactose, making it an excellent resource for biotechnological valorization [24,25]. Integrating dark fermentation and photofermentation, with *R. palustris*, offers a sustainable method to treat SCW, reducing COD and producing bio-H_2_ and/or PHB as valuable by-products.

Lactic acid bacteria (LAB) are widely used in the fermentation of dairy products due to their ability to metabolize carbohydrates into organic acids, exopolysaccharides, and other compounds, depending on the type of fermentation they perform (homo- or heterofermentative). LAB can be used to convert residual lactose present in SCW into lactate, which is the preferred carbon source for *R. palustris* in the photobiological production of bio-H_2_ [26,27].

The efficiency of bio-H_2_ and PHB production in PNSB depends on the quality of light, as bacteriochlorophylls (BChls) absorb light at 590 nm and in the near-infrared range (800–880 nm) [28]. Studies show that LEDs matched to BChl absorption significantly enhance bio-H_2_ production [29]. For instance, *R. palustris* achieved higher H_2_ yields under 590 nm light compared to other wavelengths [30], while filtering light above 760 nm reduced H_2_ production in *Rhodobacter sphaeroides* [31].

This study evaluates the potential for producing Bio-H_2_ and PHB from SCW using *R. palustris* strain 42OL in a two-stage process within a 5 L bioreactor. The first step involves lactic fermentation to enrich the substrate with lactic acid, followed by photofermentation under two types of LED lighting: white LEDs (WL) and selected LEDs (SL) emitting in the yellow (593 nm) and infrared (860 nm) ranges. The aim is to assess the feasibility of SCW valorization while analyzing the effects of light on *R. palustris* growth, BChl production, bio-H_2_ generation, and PHB synthesis.

## 2. Materials and Methods

### 2.1. Bacterial Strains

For the dark fermentation tests, a microbial consortium consisting of two different species of lactic acid bacteria (LAB) was used: *Lactococcus lactis* MK L84 and *Lacticaseibacillus paracasei* MK L49, both belonging to the collection of Department of Agriculture, Food, Environment and Forestry (DAGRI) of the University of Florence (Italy) and previously isolated from spontaneous raw milk fermentations [32]. The selected LAB strains were chosen for their proven fermentative efficiency, robustness, and ability to produce lactic acid from complex substrates, as reported by Galli et al. [32].

For the photofermentation tests, *Rhodopseudomonas palustris* 42OL was employed, a microorganism from the DAGRI collection isolated in 1973 from a waste treatment pond of a sugar refinery. This strain was selected due to its adaptability to different types of substrates and because it has been reported in the literature to achieve high bio-H_2_ production yields [33]. The draft genome sequence of *R. palustris* 42OL is available in the NCBI database under BioProject accession number PRJNA283573 (assembly accession GCA_001020905.1).

### 2.2. Cultivation Media

Before being used in the dark fermentation tests, the lactic acid bacteria (LAB) were cultured for 48 h in M17 broth (Oxoid, Basingstoke, UK) with the following composition: Tryptone 5.0 g L^−1^; Soy Peptone 5.0 g L^−1^; Yeast Extract 2.5 g L^−1^; Meat Extract 5.0 g L^−1^; Sodium Glycerophosphate 19.0 g L^−1^; Magnesium Sulphate 0.25 g L^−1^; Ascorbic Acid 0.5 g L^−1^; Lactose 5.0 g L^−1^; deionized water. The pH of the medium was 6.69 ± 0.2 at 25 °C. The strains were maintained at 30 °C under constant agitation (80 rpm). Stationary phase cells were harvested, centrifuged at 3700 rpm for 15 min (Sigma Laborzentrifugen GmbH, Osterode am Harz, Germany), and resuspended in the SCW for tests. The dark fermentation process of SCW will be explored in detail in Section 2.4.

The culture of *R. palustris* 42OL was maintained at 30 °C, under a light intensity of 150 µmol (photons) m^−2^ s^−1^, in an RPN medium, containing DL-malic acid as the primary carbon source [34]. The medium had the following composition: DL-malic acid 2.0 g L^−1^; NH_4_Cl 0.5 g L^−1^; K_2_HPO_4_ 0.5 g L^−1^; KH_2_PO_4_ 0.3 g L^−1^; MgSO_4_·7H_2_O 0.4 g L^−1^; NaCl 0.4 g L^−1^; CaCl_2_·2H_2_O 0.075 g L^−1^; Ferric citrate 0.005 g L^−1^; Yeast Extract 0.4 g L^−1^. Trace elements were provided by adding 10 mL per liter of a solution containing: ZnSO4·7H_2_O 10 mg L^−1^; MnCl_2_·4H_2_O 3 mg L^−1^; H_3_BO_3_ 30 mg L^−1^; CoCl_2_·6H_2_O 20 mg L^−1^; CuCl_2_·2H_2_O 1 mg L^−1^; NiCl_2_·6H_2_O 2 mg L^−1^; Na_2_MoO_4_·2H_2_O 30 mg L^−1^. The pH of the medium was adjusted to 6.8 with NaOH before autoclaving.

For bio-H_2_ production tests, *R. palustris* 42OL was activated in RPP medium for 10 days, as described by Bianchi et al. [34]. The strain was maintained at 30 °C, under a light intensity of 150 µmol (photons) m^−2^ s^−1^. The medium had the following composition: DL-lactic acid 3.6 g L^−1^; Na glutamate 1 g L^−1^; K_2_HPO_4_ 0.5 g L^−1^; KH_2_PO_4_ 0.3 g L^−1^; MgSO_4_·7H_2_O 0.4 g L^−1^; NaCl 0.4 g L^−1^; CaCl_2_·2H_2_O 0.075 g L^−1^; Ferric citrate 0.005 g L^−1^. The trace elements were added as reported above. To promote microbial growth, 1 mL per liter of medium of a solution containing 20 mg per 100 mL of p-aminobenzoic acid (PABA) was added after autoclave. The pH of the medium was adjusted to 6.8 with NaOH before autoclaving. Activated cells were then centrifuged for 20 min at 5000 rpm (Beckman Coulter, Brea, CA, USA) and resuspended in the SCW for the photofermentation tests.

### 2.3. Bioreactor Design

**Figure 1:**
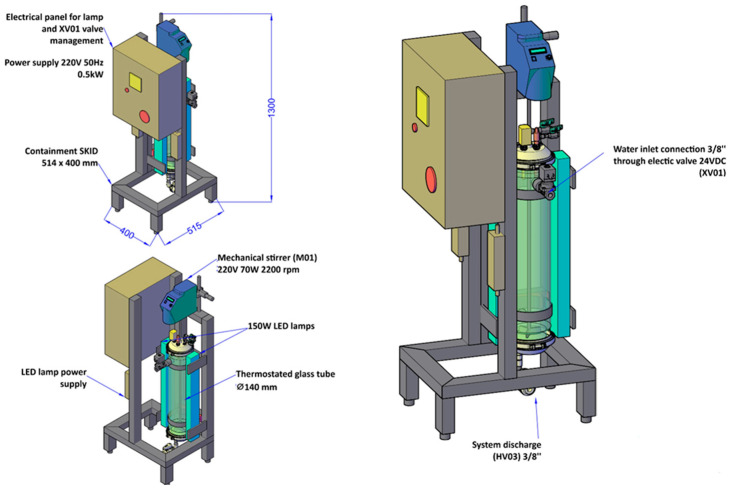
Layout of the bioreactor for the growth of PNSB used in this study.

The photofermentation test was set up in a tubular photobioreactor designed by Biosyntex S.r.l. (Bologna, BO, Italy) and constructed by Tecnocom S.r.l. (Prato, PO, Italy). The layout of the bioreactor used in this study is reported in Figure 1. The system consists of a skid of dimensions 515 mm × 400 mm, comprising: (1) a thermostated DURAN glass tube, measuring H 700 mm × D 140 mm and equipped with an internal chamber of D 110 mm; (2) a pH control instrument (B&C Electronics S.r.l., Carnate, MB, Italy); (3) a temperature control instrument (Gefran S.p.a., Provaglio d’Iseo, BS, Italy); (4) a mechanical stirrer model AM20-D 50 (Argolab, Carpi, MO, Italy) with manual speed control and a polypropylene (PP) rod (H 800 mm × D 8 mm); (5) two 150 W dimmable LED lamps, measuring 600 mm × 70 mm × 40 mm (Ambra Elettronica S.r.l., Bolzano Vicentino, VI, Italy); (6) an ON/OFF electric valve for thermostatic control; and (7) an electrical control and command panel.

The bioreactor makes it possible to replace white LED lamps (WL) with selected LED lamps (SL). The WL emitted light across different wavelengths with the following distribution: 19.66% in the blue region (λ = 450 nm), 15.20% in the green region (λ = 520 nm), 64.19% in the red region (λ = 660 nm), and 0.96% in the far-red region (λ = 730 nm). The SL emitted in the yellow region (λ = 593 nm) and the infrared region (λ = 860 nm), with distributions of 80% and 20%, respectively.

### 2.4. Dark Fermentation of SCW

This study used a SCW obtained from ricotta cheese production. The effluent was provided by the cheese factory “I Formaggi del Dottore”, located in Castelfiorentino (FI), Italy. The effluent was stored at −20 °C until use. The SCW was initially analyzed for its macro- and microelement content, as well as its ammonium, lactose, and lactic acid content. Further details on the analytical methods employed will be provided in Section 2.6.

The SCW was initially tested to evaluate potential lactic acid production through self-fermentation, using its indigenous microflora. The tests were conducted in triplicate. Briefly, 40 mL of SCW, diluted at 25% *v*/*v* with non-sterile deionized water, were incubated at 30 °C for 96 h under constant agitation at 80 rpm in tightly sealed 50 mL Falcon^®^ tubes. Lactic acid production was quantified at the end of the experiment using High-Performance Liquid Chromatography (HPLC) analysis (see Section 2.6)

To test the SCW as a substrate for lactic acid production, a co-inoculation was performed using the LAB microbial consortium described in Section 2.1. The inocula of the two LAB species were prepared as outlined in Section 2.2 and co-resuspended in 40 mL of defrosted SCW, diluted at 25% *v*/*v* with non-sterile deionized water, to a final concentration of 1 × 10^8^ CFU mL^−1^ for each strain. The test was conducted in triplicate, using tightly sealed 50 mL Falcon^®^ tubes, and incubated under the same conditions described above. Lactic acid production was quantified at the end of the experiment through HPLC analysis (see Section 2.6).

After the preliminary tests in tightly sealed 50 mL Falcon^®^ tubes, the dark fermentation experiments were continued inside the 5-litre bioreactor, as described below.

The SCW was diluted at 25% *v*/*v* with non-sterile deionized water. During the dilution, 10 mL L^−1^ of a phosphate-buffered solution was added, with the following composition: K_2_HPO_4_ 50 g L^−1^; KH_2_PO_4_ 30 g L^−1^. The LAB strains were co-inoculated into the effluent at a final concentration of 1 × 10^8^ CFU mL^−1^ each.

Dark fermentation was conducted at 30 °C, with continuous stirring at 60 rpm, and lasted for 96 h. Three different pH conditions were evaluated to identify the optimal conditions for achieving the highest lactic acid yields: without pH control, pH maintained between 3.5 and 4.5, and pH maintained between 4.5 and 5.5. The pH was corrected using NaOH 4 M. The experiments were performed in duplicate for each pH condition. The lactic acid content was monitored every 24 h.

### 2.5. Photofermentation Tests

For the photofermentation trials conducted with *R. palustris* 42OL, the dark-fermented SCW obtained with controlled pH (maintained between 3.5 and 4.5, and between 4.5 and 5.5) was combined, homogenized, and used as a culture medium. The effluent was further diluted with non-sterile deionized water at a final concentration of 18% *v*/*v* (compared to undiluted SCW) and supplemented with 10 mL L^−1^ of the following solutions: phosphate buffer (K_2_HPO_4_, 50 g L^−1^; KH_2_PO_4_, 30 g L^−1^), ferric citrate (0.5 g L^−1^), and MgSO_4_·7H_2_O (40 g L^−1^), as described by Adessi et al. [26]. The culture medium was autoclaved twice at 121 °C for 15 min, with a 48 h interval between the two cycles, to ensure the complete elimination of LAB. The pH was adjusted to 6.8 with 2 M NaOH before and after each autoclaving. Between the two autoclaving cycles, the culture medium was centrifuged under sterile conditions at 5000 rpm for 15 min (Beckman Coulter, Brea, CA, USA). This was followed by sterile filtration through filter paper under a horizontal laminar flow hood.

*R. palustris* 42OL, previously grown in RPP medium (described in Section 2.2) was inoculated into the culture medium, in a final volume of 5.2 L. The initial cell concentration was equal to OD_660nm_ = 0.4 for each test.

Two distinct lighting conditions were tested for the culture: white LED lights (hereafter referred to as WL) and selected LED lights (hereafter referred to as SL). Detailed information about the lights used in these tests is provided in Section 2.3.

The experiments were conducted in duplicate for each type of light, with a total duration of 336 h for each test. The culture was initially exposed to a light intensity of 150 µmol photons m^−2^ s^−1^ for the first 24 h to allow the microorganism to adapt to the bioreactor environment. This intensity was then increased to 300 µmol photons m^−2^ s^−1^ until 168 h after the start of the experiment and finally increased to 400 µmol photons m^−2^ s^−1^ for the remainder of the experiment. The adjustment in light intensity was necessary to ensure optimal photosynthetic activity. Indeed, the growth of the microorganism, with the consequent increase in the medium’s turbidity, limited light penetration in the bioreactor.

Bacterial growth was assessed by measuring bacteriochlorophyll *a* (BChl*a*), optical density at 660 nm (OD_660nm_) every 48 h, and cellular dry weight (CDW) at 0, 168, and 336 h from the start of the experiment. Lactic acid consumption was measured every 48 h from the start of the experiment, while ammonium (NH_4_^+^) concentration was determined at 0, 168, and 336 h from the beginning of the experiment. PHB production was determined at the end of the experiment (336 h). Further details on the analytical methods used are provided in Section 2.6.

The bio-H_2_ gas produced was collected on a calibrated column and measured by the water displacement method [26]. The calibrated column was submerged in a CO_2_-absorber solution (1.0 M NaOH, 3.4 M NaCl), as reported by Touloupakis et al. [35].

### 2.6. Analytical Methods

The lactic acid concentration was determined by High-Performance Liquid Chromatography (HPLC) analysis (Varian Inc., Palo Alto, CA, USA). Before injection, each sample was centrifuged at 14,000 rpm for 10 min to ensure the complete separation of solid particles from the supernatant. Separation was obtained with a Rezex ROA organic acid H+ column (300 mm × 7.8 mm; Phenomenex, Castel Maggiore, Bologna, Italy), connected to a refractive index detector (Knauer K-2301, GmbH, Berlin, Germany) and UV detector (λ = 210). Elution was performed at 65 °C with 0.013 N H_2_SO_4_ eluent at a flow rate of 0.6 mL/min. Data were collected and analyzed by using the Galaxie software version 1.8.4.1 (Varian Inc., Palo Alto, CA, USA). Quantitative analysis was carried out by standard curve designed for lactic acid.

The concentration of metals and other inorganic compounds was determined through an inductively coupled plasma-optical emission spectrometry ICP-OES analyzer (Thermo Scientific™ iCAP™ 7400, Waltham, MA, USA), using IRSA 3010 (conventional acid mineralization) and IRSA A-3020 (determination of chemical elements by spectroscopy emission with plasma source) methods.

CDW was determined in triplicate on a 3 mL culture sample, through a filtration with 0.45 μm Mixed Cellulose Esters (MCE) membranes (Fisher Scientific International, Inc., Pittsburgh, PA, USA). Membranes were previously activated with 5 mL of distilled water. After filtration the cells were washed twice with distilled water, oven-dried at 105 °C for 3 h, and weighed on an analytical balance (Sartorius AG, Göttingen, Germany).

BChl*a* content was determined according to Carlozzi and Sacchi [36]. Briefly, a 2 mL sample was centrifugated at 7000 rpm for 10 min with a Hermle Z 167 M centrifuge (Hermle Labortechnik GmbH, Wehingen, Germany), and the supernatant was removed. BChl*a* was extracted from the cell pellet, resuspending it with a 2 mL acetone/methanol 7:2 *v*/*v* ratio solution. The sample was incubated at 4 °C for 30 min and centrifugated at 7000 rpm for 10 min. BChl*a* content was determined by measuring the absorbance (A) at 775 nm (ɛ = 75 mM^−1^ cm^−1^) with a Varian Cary50 UV-visible spectrophotometer (Varian, Mulgrave, Australia).

The optical density was determined in triplicate on 2 mL samples at a wavelength of 660 nm (OD_660nm_) using a Varian Cary50 UV-visible spectrophotometer (Varian, Mulgrave, Australia). In the spectrophotometric measurement, the DF-SCW was used as the blank.

The cellular concentration of lactic acid bacteria (LAB) for determining the inoculum in dairy wastewater was measured using cell counting with a Neubauer improved counting chamber (Paul Marienfeld GmbH & Co. KG, Lauda-Königshofen, Germany) with a depth of 0.01 mm.

The ammonium concentration was determined using the HI93764B-25 Ammonia HR reagents set (Hanna Instruments, Woonsocket, RI, USA), based on the Nessler method, and quantified spectrophotometrically using a Varian Cary50 UV-visible spectrophotometer (Varian, Mulgrave, Australia), as reported by Rice et al. [37].

The intensity of the light reaching the culture surface was measured with a quantum/radiometer/photometer model DO9721 equipped with a quantum sensor model LP9021 (Delta Ohm, Padua, Italy).

Bio-H_2_ was collected and quantified volumetrically using the water displacement method, as described in Section 2.5, and subsequently analyzed by gas chromatography, as reported by Adessi et al. [38].

Poly-β-hydroxybutyrate (PHB) production was determined at the end of the experiment (336 h), according to the method described by De Philippis et al. [39].

### 2.7. Calculation and Statistical Analysis

The substrate conversion efficiency (SCE) was determined according to Equation (1) [28], as the percentage ratio between the moles of hydrogen produced and the theoretical moles of hydrogen obtainable from the complete conversion of the substrate into hydrogen (Equation (2)) [26].

(1) $$SCE\;\left(\%\right)=\frac{molH_{2}\;obtained}{molH_{2}\;theoretical}\times 100$$

C_3_H_6_O_3_ + 3H_2_O → 6H_2_ + 3CO_2_ (2)

The moles of hydrogen produced were calculated based on the hydrogen collected, applying the ideal gas law under ambient conditions (T = 25 °C, P = 1 atm).

The efficiency of light energy conversion to hydrogen (LCE) during the hydrogen production period was calculated according to Equation (3), as Adessi et al. [26] reported, assuming that the culture absorbed all the incident light.

(3) $$LCE\;\left(\%\right)=\frac{Combustion\;enthalpy\;of\;H_{2}\times H_{2}\;production\;rate}{Absorbed\;light\;energy}\times 100\;$$

All analyses were conducted in experimental triplicates (*N* = 3); a minimum number of three instrumental replicates was always used for each measurement (*n* = 3). To determine whether the results were significantly different, the data were analyzed using one-way analysis of variance (ANOVA) at the 95% level of significance, followed by Tukey’s post hoc test of honest difference in significance (HSD). The results were considered statistically significant at *p* ≤ 0.05. Before the ANOVA, all data were checked for assumptions of normality using the D’Agostino-Pearson normality test and for homogeneity of variance using Bartlett’s test. To check for statistical differences between the mean values of the two lighting conditions, independent *t*-tests were performed. All analyses were conducted using R software version 4.4.1.

## 3. Results

### 3.1. Chemical Composition of SCW

The chemical composition of SCW used in this study (before dilution) is reported in Table 1. The effluent had an initial pH of 6.30 (±0.02).

The analysis revealed that lactose was the most abundant compound, with a concentration of 43.73 g L^−1^ (±1.72). Lactic acid was detected in lower amounts, at 0.31 g L^−1^ (±0.08). Regarding the nitrogen content, ammonium (NH_4_^+^) was present at a significant concentration of 504.53 mg L^−1^ (±7.47).

Among the macronutrients, sodium (Na), phosphorus (P), and potassium (K) were present in the highest concentrations, with values of 0.88 g L^−1^ (±0.06), 0.31 g L^−1^ (±0.03), and 1.66 g L^−1^ (±0.15), respectively. Additionally, calcium (Ca), magnesium (Mg), and sulfur (S) were present in measurable concentrations, with values of 0.23 g L^−1^ (±0.02), 0.16 g L^−1^ (±0.015), and 7.83 mg L^−1^ (±1.37), respectively.

As regards the micronutrients, boron (B) was present at the highest concentration, 0.26 (±0.02) mg L^−1^, followed by iron (Fe) and zinc (Zn), with concentrations of 0.25 (±0.11) and 0.13 (±0.05) mg L^−1^, respectively. Finally, trace elements including cobalt (Co), copper (Cu), manganese (Mn), molybdenum (Mo), nickel (Ni), and vanadium (V) were detected at microgram-per-liter levels.

### 3.2. Lactic Acid Production During Dark Fermentation of SCW

The first step assessed whether the indigenous microflora in the defrosted and diluted SCW could produce significant amounts of lactic acid. After 96 h of testing, the lactic acid concentration reached 0.74 g L^−1^ (±0.07), with a final substrate pH of 4.91 (±0.07). To maximize lactic acid production, fermentation was evaluated by co-inoculating two LAB species (see Section 2.1 for further details on the species used). In this case, after 96 h, the lactic acid concentration reached 2.06 g L^−1^ (±0.09), with a final pH of 3.37 (±0.11). An independent *t*-test revealed a statistically significant difference in lactic acid production between the two conditions (without inoculum and with co-inoculum) (*p* < 0.001, ***), confirming that the inoculated samples produced significantly more lactic acid compared to the non-inoculated ones.

Subsequent efforts focused on optimizing the dark fermentation process in a 5-litre bioreactor, to maximize lactic acid yield from the co-inoculation of LAB. Three different pH conditions were tested in duplicate: (1) without pH control; (2) 3.5 ≤ pH < 4.5; and (3) 4.5 ≤ pH < 5.5.

**Figure 2:**
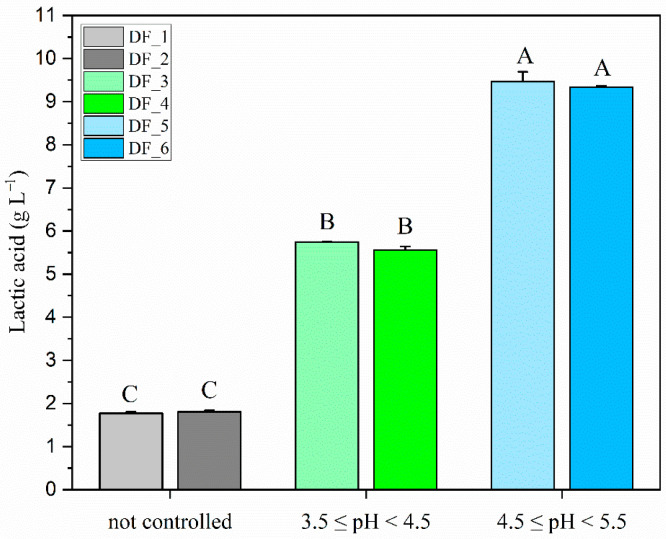
Lactic acid yield (g L^−1^) after 96 h of dark fermentation using the co-inoculation of *L. lactis* MK L84 and *L. paracasei* MK L49 under different pH conditions. Trials were conducted without pH control (DF_1 and DF_2), at pH 3.5 ≤ pH < 4.5 (DF_3 and DF_4), and at pH 4.5 ≤ pH < 5.5 (DF_5 and DF_6). Error bars indicate the standard deviation of 3 technical replicates. Different letters above the bars indicate statistically significant differences according to one-way ANOVA (95% significance level) followed by Tukey’s HSD post hoc test.

In the tests without pH control, the pH dropped to 3.53 (±0.05) in the first 24 h, reaching a final value of 3.14 (±0.04) at the end of the experiment. Figure 2 shows the lactic acid yield (g L^−1^) after 96 h post-inoculation for the three conditions tested, with statistically significant differences (*p* < 0.001, ***). Lactic acid production remained very low in dark fermentation (DF) trials DF_1 and DF_2 without pH control, reaching an average concentration of 1.75 g L^−1^ (±0.00). Lactic acid production increased significantly to an average value of 5.53 g L^−1^ (±0.09) under controlled pH 3.5 ≤ pH < 4.5 in DF_3 and DF_4 tests. The highest production was achieved in the pH range of 4.5 ≤ pH < 5.5 (DF_5 and DF_6), with an average lactic acid concentration of 9.35 g L^−1^ (±0.11).

### 3.3. Photofermentation Results

Table 2 presents the substrate’s initial ammonium and lactic acid concentrations for each photofermentation test. C/N ratios are also reported.

Concerning the initial ammonium content, variability was observed among different photofermentation runs. The WL_1 and WL_2 runs showed similar ammonium levels, with no statistically significant differences, and mean concentrations of 90.50 mg L^−1^ (±2.20) and 91.60 mg L^−1^ (±1.90), respectively. In contrast, the SL_1 run had a significantly higher initial ammonium content (*p* < 0.01, **) compared to SL_2, with concentrations of 103.27 mg L^−1^ (±8.76) and 75.73 mg L^−1^ (±2.23), respectively. No statistically significant differences were observed among the four photofermentation runs concerning lactic acid content (*p* = 0.203).

**Figure 3:**
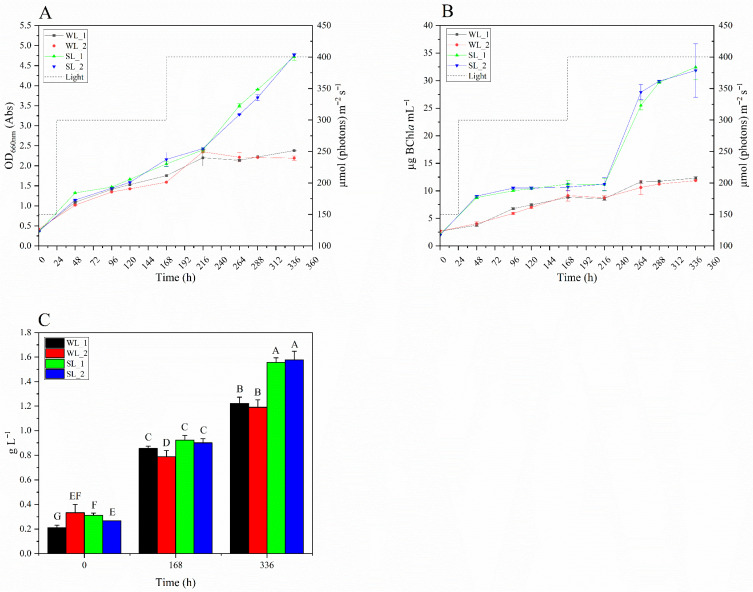
(**A**) Growth of *R. palustris* 42OL, represented as OD_660nm_, under WL and SL conditions during photofermentation. (**B**) BChl*a* accumulation, under WL and SL conditions over 336 h. (**C**) Variation in the CDW of *R. palustris* 42OL over time under WL and SL conditions. The light intensity is reported as a dashed line (- - -). Error bars indicate the standard deviation of 3 technical replicates. Different letters above the bars indicate statistically significant differences according to one-way ANOVA (95% significance level) followed by Tukey’s HSD post hoc test.

The growth of *R. palustris* 42OL in each of the four photofermentation runs was monitored by measuring the CDW at 0, 168, and 336 h (Figure 3C), as well as every 48 h by assessing the OD_660nm_ (Figure 3A) and quantifying BChl*a* concentration (Figure 3B). The light intensity was managed in three steps during the experiment. It was initially set to 150 µmol m^−2^ s^−1^ for the first 24 h, then increased to 300 µmol m^−2^ s^−1^ for the following 144 h, and finally raised to 400 µmol m^−2^ s^−1^ for the remaining 168 h.

In all four runs, a progressive increase in CDW (Figure 3C) was observed over time, with the highest values recorded at the end of the experiment in the two tests conducted under SL conditions. Across all conditions, the most substantial increases occurred between 168 and 336 h, coinciding with an increase in light intensity from 300 to 400 µmol m^−2^ s^−1^, except for WL_2, where a slightly larger increase was observed between 0 and 168 h. The greatest overall increase in CDW was observed in the SL_2 test, with a rise of +0.68 g L^−1^ between 168 and 336 h. After 336 h post-inoculation, statistically significant differences in CDW were observed between the WL and SL runs.

Figure 3A illustrates the variation in OD_660nm_ during the 336 h test period for the four runs. Up to 216 h, all tests showed a progressive and comparable growth of OD_660nm_. After this point, OD_660nm_ stabilized in the two WL runs, reaching final values of 2.38 (±0.06) for WL_1 and 2.19 (±0.06) for WL_2. In contrast, the SL runs showed a continuous growth of OD_660nm_, with final values of 4.72 (±0.09) for SL_1 and 4.76 (±0.04) for SL_2. Notably, as with the CDW measurements, the divergence between the WL and SL curves became evident after the second increase in light intensity, from 300 µmol m^−2^ s^−1^ to 400 µmol m^−2^ s^−1^.

Figure 3B presents the accumulation of BChl*a* over time for the four photofermentation runs. The trend closely mirrors OD_660nm_, with a pronounced increase in BChl*a* concentration in the SL tests after 216 h, following the second increase in light intensity. During the first 216 h, BChl*a* levels remained consistently higher in the SL trials than in the WL trials. At 336 h BChl*a* levels in SL_1 and SL_2 peaked, respectively, at 32.45 µg mL^−1^ (±2.29) and 31.84 µg mL^−1^ (±4.84), significantly higher than WL_1 (12.32 µg mL^−1^ ± 0.26) and WL_2 (11.85 µg mL^−1^ ± 0.13) (*p* < 0.001, ***).

**Figure 4:**
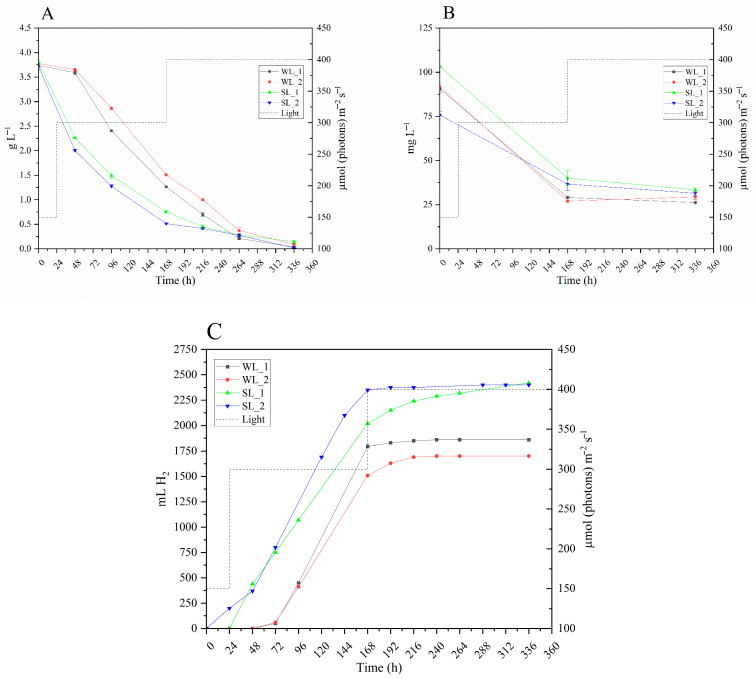
(**A**). Lactic acid concentration (g L^−1^) under WL and SL conditions during 336 h of photofermentation. (**B**) Ammonium consumption (mg L^−1^) over 336 h under WL and SL conditions. (**C**) Time course of bio-H_2_ production (mL) under WL and SL conditions during 336 h of photofermentation. The light intensity is reported as a dashed line (- - -). Error bars indicate the standard deviation of 3 biological replicates.

Figure 4A shows the progression of lactic acid concentration during 336 h for the four photofermentation tests. In all tests, the lactic acid concentration reaches values close to zero at the end of the experiment. However, in the SL tests, consumption was significantly faster in the first two days after the start of the experiment. Specifically, the percentage consumption in the first 48 h was −40.53% in SL_1 and −46.05% in SL_2, whereas in WL_1 and WL_2 it was only −4.01% and −3.27%, respectively.

Figure 4B illustrates the progression of NH_4_^+^ concentration over time for the four runs. During the first 168 h, all runs showed significant reductions in NH_4_^+^ levels, with rates varying between conditions. More specifically, the WL runs showed the greatest reductions, with WL_1 showing a reduction of 67.90% and WL_2 the highest at 70.47%. In contrast, the SL runs showed smaller decreases, with SL_1 reducing by 61.18% and SL_2 by 51.56%. After 168 h, the NH_4_^+^ consumption slowed across all runs, coinciding with the increase in light intensity from 300 to 400 µmol photons m^−2^ s^−1^, reaching similar levels by the end of the experiment.

The final bio-H_2_ production obtained at the end of the experiment, for the four runs, is shown in Table 3, and the time course of bio-H_2_ production is reported in Figure 4C. The time course of bio-H_2_ production varied significantly across the four runs. In the WL_1 and WL_2 conditions, bio-H_2_ production began only after 48 h, with WL_1 stabilizing at 1860 mL by 168 h, and WL_2 reaching 1700 mL by 192 h, with no significant increases afterwards. In contrast, the SL_1 condition started bio-H_2_ production at 24 h, reaching a maximum value of 2420 mL at 336 h. The SL_2 condition showed the most rapid onset, with production starting immediately at the beginning of the experiment and reaching 2400 mL by 288 h, when it stabilized. At the end of the runs, the SL condition showed a significantly higher total bio-H_2_ production (*p* < 0.05, *) than the WL condition.

Regarding the VHPR and the specific hydrogen production rate (SHPR), shown in Table 3, no statistical difference was observed between the two types of illumination.

Table 3 also shows data on light utilization efficiency (LCE) and substrate (lactic acid) to H_2_ conversion efficiency (SCE). While no statistically significant differences were recorded for the first parameter between the two lighting types, the SCE was significantly higher (*p* < 0.05, *) in the SL tests.

Table 3 reports the PHB production at the end of the experiment for the four photofermentation runs. *R. palustris* 42OL accumulated significantly higher levels of PHB (% *w*/*w*) in the trials conducted under selected light (SL) compared to those under white light (WL) (*p* < 0.001, ***). In the SL trials, PHB levels reached 1.65% (±0.03) and 1.67% (±0.03) for SL_1 and SL_2, respectively.

## 4. Discussion

The dairy industry produces organic-rich wastewater that, if discharged untreated into water bodies, causes acidification and eutrophication, harming ecosystems. Using PNSB to convert this wastewater into bio-H_2_ and PHB offers a solution, reducing organic load while generating valuable compounds.

The analysis of the raw composition of SCW used in this study revealed a high NH_4_^+^ concentration (504.53 mg L^−1^ ± 7.47). The literature widely documents that an excess of ammoniacal nitrogen inhibits bio-H_2_ production by PNSB, as it interferes with the regulatory mechanisms of the nitrogenase enzyme [40,41]. For this reason, the effluent was initially diluted at 25% *v*/*v* with non-sterile deionized water to significantly reduce ammonium content while retaining enough lactose for the subsequent dark fermentation step. Although dilution increases substrate volume and management costs, it enhances light penetration in the photofermentation phase, thus shortening the bio-H_2_ accumulation lag phase [14,15].

Lactate is the preferred carbon source for *R. palustris* 42OL to maximize bio-H_2_ yield [42]. The lactate content in the raw SCW (0.31 g L^−1^ ± 0.08) was approximately one order of magnitude lower than in the synthetic RPP medium (3.6 g L^−1^). The subsequent dilution of the effluent further reduced the lactose concentration, necessitating a dark fermentation step to achieve lactate enrichment.

To select the best conditions to produce lactate with a minimum number of treatments, we initially considered using the effluent’s indigenous microflora to ferment lactose into lactate. However, trials yielded only a minimal increase in lactate, reaching a final concentration of 0.74 g L^−1^ (±0.07), still substantially below the level in the RPP synthetic medium. This limited lactic acid production was probably due to the freezing process applied to the effluent, which may have altered and reduced the indigenous microflora. Therefore, we investigated the effect of a LAB co-inoculum, which resulted in a significantly higher lactic acid yield of 2.06 g L^−1^ (±0.09), nearly tripling the yield obtained by the indigenous microflora. However, this lactate concentration was still insufficient for photofermentation, remaining below that of the synthetic RPP medium. At the end of the LAB co-inoculation tests, the pH reached 3.37 (±0.11), likely limiting lactate yield, as its production is minimal in acidic conditions [43]. Therefore, our focus shifted towards identifying the optimal substrate pH to maximize lactate production by LAB co-inoculum in a 5 L photobioreactor. To prevent the pH from dropping too quickly and complicating manual control, a phosphate buffer was added to the substrate (see Section 2.4). The highest yields were achieved at pH between 4.5 and 5.5 (Figure 2), with an average lactate concentration of 9.40 g L^−1^ (±0.09), which was 1.7 and 5.2 times higher than the results obtained at pH levels between 3.5 and 4.5 and with uncontrolled pH, respectively. This result is supported by studies showing that the optimal pH for lactic acid production lies between 5 and 6, where LAB activity peaks, enabling efficient fermentation [43,44,45]. Moradi et al. [46] produced 14.2 g L^−1^ lactic acid using *Lactobacillus delbrueckii* subsp. *lactis* (PTCC: 1743) on a substrate consisting of DW and glucose, with a fermentation time of 41.41 h at 37 °C and an enzymatic hydrolysis step. Our co-inoculum produced approximately 34% less lactic acid, but with significant advantages that simplify the process and reduce overall costs: reduced production time, a lower temperature (30 °C), and the absence of expensive carbon sources or additional enzymatic treatments.

Prior to each bio-H_2_ production experiment, the photofermentation substrate was supplemented, as nutrient levels were insufficient due to prior dilution. For this purpose, iron citrate and magnesium sulfate were added, based on Adessi et al. [26], who showed these nutrients enhance bio-H_2_ production by *R. palustris* 42OL grown on fermented bread effluent. Iron and sulfate are essential for nitrogenase synthesis [38], while magnesium is involved in BChls synthesis [47].

Growth, bio-H_2_, and PHB production in *R. palustris* 42OL were then evaluated on the dark-fermented SCW, comparing white light (WL) LEDs with specific wavelength LEDs (SL, 593 nm, and 860 nm) matching the absorption peaks of BChls in *R. palustris* 42OL. This approach clarified how specific wavelengths enhance photofermentative efficiency and high-value molecule yields.

Light intensity was gradually raised throughout the experiment to maintain adequate light penetration as cell density increased. Initially, it was set to 150 µmol photons m^−2^ s^−1^ for the first 24 h to minimize cellular stress and support cell adaptation. It was then increased to 300 µmol photons m^−2^ s^−1^ for the following 144 h and finally elevated to 400 µmol photons m^−2^ s^−1^ until the end of the experiment.

The first bio-H_2_ production experiment in the 5 L photobioreactor under white light (WL), using substrate diluted at 25% (*v*/*v*), showed no gas accumulation during the first 168 h post-inoculation. This result was attributed to the presence of ammonium at concentrations exceeding inhibitory levels for *R. palustris* 42OL.

The presence of ammonium ions above a threshold concentration of approximately 2 mM (corresponding to approximately 36 mg L^−1^ NH_4_^+^) has been reported to inhibit bio-H_2_ production by PNSB. However, this inhibitory threshold may vary depending on the specific PNSB strain and operating conditions [48]. Consequently, to reduce ammonium inhibition while simultaneously limiting substrate dilution and water consumption, the dark-fermented substrate was diluted to achieve an ammonium concentration below 100 mg L^−1^.

The substrate diluted to this level (18% *v*/*v* relative to the raw wastewater) was used in the subsequent four photofermentation experiments. The initial lactate and ammonium concentrations for each photofermentation test are reported in Table 2.

The cumulative bio-H_2_ production trends under WL and SL conditions are shown in Figure 4C. In the WL runs, bio-H_2_ production began 48 h after inoculation. On the contrary, the SL runs showed a reduced lag phase; in SL_1, bio-H_2_ production started after 24 h, while in SL_2, it began immediately. The difference in lag phases between the two SL trials is likely connected to the lower ammonium concentration in SL_2 compared to SL_1, due to the heterogeneity of the substrate.

Table 3 shows the total bio-H_2_ and PHB production at the end of the experiment. On average, the SL condition produced 35.5% more H_2_ than the WL condition. Similarly, PHB production increased significantly in the SL condition, almost tripling the values obtained with WL.

The higher production of H_2_ and PHB under selected light wavelengths is likely due to higher light utilization efficiency. BChl*a*, in PNSB, has absorption peaks around 590–600 nm and 850–870 nm [28], making selected wavelengths more effective than broad-spectrum WL. Targeted light absorption enhances photophosphorylation, generating ATP and reducing power (NADPH) essential for PHB and H_2_ biosynthesis [21]. In contrast, exposure to broad-spectrum light may increase energy dispersion and photo-oxidative stress due to inefficiently absorbed wavelengths.

The higher efficiency of the SL compared to WL is evident also in the growth profiles of *R. palustris* 42OL (Figure 3). During the first 168 h, growth proceeds similarly in both lighting conditions, with a steady increase in CDW, OD_660nm_, and BChl*a* levels over time. Under SL conditions, a higher lactic acid consumption is observed (Figure 4A), mainly due to the higher biomass concentration achieved under SL, since the specific H_2_ production rates are the same (not significantly different) under SL and WL conditions (Table 3). After 168 h, the increase in light intensity from 300 to 400 µmol photons m^−2^ s^−1^ marked a critical point: bio-H_2_ production decreased drastically (Figure 4C), and ammonium consumption stabilized (Figure 4B). This is followed by a 48 h phase in which both lighting conditions show a steady and comparable increase in OD_660nm_, along with stabilization in BChl*a* synthesis, likely as an adaptation to the new light conditions. From 216 h onwards, the conditions diverge markedly; under SL, a significant increase in BChl*a* and OD_660nm_ is observed, likely due to greater efficiency in photon utilization under this condition. In contrast, WL leads to high energy dissipation and photooxidative stress, halting growth and limiting BChl*a* accumulation. Despite sustained cellular growth under SL, ammonium consumption stabilization appears contradictory, as nitrogen is essential for building cellular biomass. However, the Nessler method used for its quantification (see Section 2.6) detects only free ammonium, not nitrogen in amino groups of amino acids [49]. Dairy wastewaters contain protein and amino acid residues that *R. palustris* 42OL can use as a nitrogen and carbon source [17,24]. The increase in light intensity, with the consequent greater availability of energy, may have favored the use of amino acids, which provide a combined source of nitrogen and carbon, advantageous under high-energy conditions. Indeed, amino acid degradation requires additional metabolic pathways and specialized enzyme systems that consume substantial amounts of ATP, helping to stabilize the cell’s energy balance. However, further studies will be needed to demonstrate the higher utilization of amino acids as a source of carbon and nitrogen under high light conditions compared to ammoniacal nitrogen.

The maximum cumulative hydrogen (H*_max_*) obtained under both lighting conditions (Table 3) was significantly lower than the value reported by Seifert et al. [50], who obtained an H*_max_* of 3.23 L H_2_ L^−1^ of substrate by cultivating *Rhodobacter sphaeroides* O.U. 001 on diluted dairy wastewater with a modified Biebel and Pfennig medium lacking a carbon source (malic acid). Optimum results were achieved at a dilution of 40% (*v*/*v*) and a light intensity of 9 klx.

It should be noted that, in the study by Seifert et al. [50], the raw dairy wastewater contained 40 mg dm^−3^ of ammonium nitrogen (N-NH_4_^+^), corresponding to approximately 51.4 mg L^−1^ of NH_4_^+^. This concentration was further reduced by dilution, as photofermentation was carried out at 40% (*v*/*v*) wastewater. Moreover, the dairy wastewater was filtered before photofermentation, which likely reduced the particulate fraction and the contribution of protein-associated nitrogen, thereby limiting ammonium availability in the photofermentation medium. Therefore, the effective ammonium concentration in their system was substantially lower than that in the present study, which employed a dark fermentation effluent.

This substantial difference in hydrogen production can be attributed not only to differences in substrate characteristics, including ammonium availability, but also to the different experimental scales, as efficiency generally decreases with process scale-up. Indeed, while Seifert et al. [50] conducted their experiments in small 25 mL vials filled to 12.5 mL, this study involved a 416-fold larger working volume (5200 mL). Furthermore, in the present study, the substrate was diluted with deionized water rather than with a synthetic medium, supplemented only with the minimal essential compounds required for hydrogen production.

Regarding PHB production, an important factor for optimizing its accumulation is the C/N ratio of the growth medium; ratios above 30 are generally recommended for optimal PHB synthesis [21]. In our study, the substrate showed an average C/N ratio of 41.8 (±3.47) (Table 2), indicating favorable conditions for PHB accumulation. However, lactate is not the preferred carbon source for PHB synthesis in PNSB; acetate and butyrate are more efficient substrates [21], but neither was detected in our substrate. Unlike lactate, which must first be oxidized to pyruvate and subsequently converted to acetyl-CoA (the key precursor for PHB synthesis), acetate and butyrate can be directly converted to acetyl-CoA with lower energetic costs. Consequently, lactate is often directed toward metabolic pathways favoring hydrogen production rather than PHB synthesis [21].

In agreement with this metabolic framework, Wu et al. [50] reported that *R. palustris* WP3-5 produced the highest cumulative H_2_ volume when grown on lactate (131.3 mL over 114 h). However, acetate exhibited the highest SCE (14.2%), indicating a more efficient conversion of the substrate into hydrogen. When lactate was used, the SCE was lower (approximately 9.3%) and remained nearly constant regardless of PHB accumulation, suggesting limited competition between PHB synthesis and H_2_ production under lactate-fed conditions.

Wu et al. [51] also reported a PHB accumulation of 1.4% (*w*/*w*) in *R. palustris* WP3-5 when grown on lactate, which is comparable to the values obtained in our study under SL conditions. In our experiments, higher PHB production was observed under SL conditions, which also exhibited a faster lactate consumption. This accelerated lactate utilization may also be attributed to the enhanced PHB synthesis observed under these conditions. However, since PHB was quantified only at the end of the experimental runs, it is not possible to assess its accumulation dynamics or potential intracellular reutilization over time.

Future experiments should therefore include measurements of PHB at different cultivation times to better elucidate its role in the distribution of reducing equivalents and its interaction with hydrogen production. This interpretation is consistent with the observations of Wu et al. [51], who suggested that PHB does not merely compete with hydrogen production for reducing power, but rather plays a more complex metabolic role, particularly under stress conditions.

## 5. Conclusions

In this study, we demonstrated the feasibility of using second cheese whey (SCW) for H_2_ and PHB production with *Rhodopseudomonas palustris* strain 42OL in a 5 L tubular photobioreactor within an integrated dark-fermentation/photofermentation system. During the dark fermentation phase, a co-inoculum of lactic acid bacteria (LAB) comprising *Lactococcus lactis* MK L84 and *Lacticaseibacillus paracasei* MK L49 proved optimal for obtaining high concentrations of lactic acid from properly diluted wastewater when maintained at 30 °C and a pH of 4.5 to 5.5 for 96 h. In the subsequent photofermentation phase, the quality of light significantly influenced the H_2_ and PHB yields. The highest yields were achieved with selected light (SL) at wavelengths corresponding to the absorption peaks of the PNSB BChls, with an average yield of 0.47 L of H_2_ per liter of substrate (±0.007) and 1.66% wPHB/wCDW (±0.01). Under SL conditions, a light intensity of 400 µmol photons m^−2^ s^−1^ appeared to favor biomass growth and BChl*a* synthesis over H_2_ production.

Dairy wastewater composition is naturally variable, mainly due to the origin of the whey and the specific production processes used to obtain it. Processes for the valorization of these effluents must be adapted accordingly. The development of strategies for the transformation of dairy waste into resources for energy and biopolymers production is crucial to converting waste streams into inputs for subsequent processes, thus supporting the transition from a linear to a circular economic model.

Future studies will focus on providing a detailed assessment of SCW conversion efficiency under the operating conditions that demonstrated the most promising performance in this study, particularly those obtained using selected lighting (SL). In addition, a comprehensive assessment of the technical and economic feasibility of the process will be necessary to determine its potential for combining biohydrogen and PHB production in the sustainable valorization of dairy wastewater. Such analyses are essential to compare this approach with alternative treatment and valorization technologies and to support its potential industrial implementation.

## Figures and Tables

**Table 1:** Chemical composition of raw SCW used in this study (before dilution). The standard deviation is indicated in brackets.

Compounds	Concentration
Lactose (g L^−1^)	43.73 (±1.72)
Lactic acid (g L^−1^)	0.31 (±0.08)
NH_4_^+^ (mg L^−1^)	504.53 (±7.47)
B (mg L^−1^)	0.26 (±0.02)
Ca (g L^−1^)	0.23 (±0.02)
Co (µg L^−1^)	0.92 (±0.32)
Cu (µg L^−1^)	19.97 (±4.97)
Fe (mg L^−1^)	0.25 (±0.11)
K (g L^−1^)	1.66 (±0.15)
Mg (g L^−1^)	0.16 (±0.015)
Mn (µg L^−1^)	11.64 (±2.45)
Mo (µg L^−1^)	13.37 (±1.53)
Na (g L^−1^)	0.88 (±0.06)
Ni (µg L^−1^)	1.23 (±0.66)
P (g L^−1^)	0.31 (±0.03)
S (mg L^−1^)	7.83 (±1.37)
V (µg L^−1^)	11.79 (±3.01)
Zn (mg L^−1^)	0.13 (±0.05)

**Table 2:** Concentrations of ammonium and lactic acid of the diluted dark-fermented SCW at the beginning of the four photofermentation tests under WL and SL conditions. The C/N ratio of each substrate in each test is also provided. Data are presented as mean values with standard deviations in brackets.

Parameter	WL_1	WL_2	SL_1	SL_2
NH_4_^+^ (mg L^−1^)	90.50 (±2.20)	91.60 (±1.90)	103.27 (±8.76)	75.73 (±2.23)
Lactic acid (g L^−1^)	3.74 (±0.05)	3.78 (±0.04)	3.80 (±0.04)	3.71 (±0.06)
C/N	41.6	41.1	38	46.4

**Table 3:** Total bio-H_2_ production, derived parameters, final PHB production, and their statistical analysis (independent two-sample *t*-test). Significance: * (*p* < 0.05), *** (*p* < 0.001), ns: not significant. SCE = substrate conversion efficiency; VHPR = volumetric hydrogen production rate; SHPR = specific hydrogen production rate; LCE = light conversion efficiency; H_max_ = maximum cumulative H2; $R_{max,H_{2}}$ = maximum rate of H_2_ production.

Parameter	WL_1	WL_2	SL_1	SL_2	*t*-Test (WL vs. SL)
SCE (%)	5.87	5.31	7.52	7.63	*
VHPR (mL L^−1^ h^−1^)	1.24	1.14	1.49	1.37	ns
SHPR (mL mg^−1^ h^−1^)	0.0064	0.0069	0.0062	0.0055	ns
Total H_2_ (mL)	1860	1700	2420	2400	*
*H_max_* (L L^−1^)	0.36	0.33	0.47	0.46	*
$R_{max,H_{2}}$ (L L^−1^ h^−1^)	0.004	0.003	0.004	0.004	ns
LCE (%)	0.14	0.13	0.11	0.12	ns
PHB (% w_PHB_/w_CDW_)	0.60 (±0.09)	0.57 (±0.05)	1.65 (±0.03)	1.67 (±0.03)	***

## Data Availability

The original contributions presented in this study are included in the article. Further inquiries can be directed to the corresponding author.

## Abbreviations

The following abbreviations are used in this manuscript:

CW	Cheese whey
DW	Dairy wastewater
SCW	Second cheese whey
Bio-H_2_	Biohydrogen
PNSB	Purple non-sulfur bacteria
CDW	Cellular dry weight
BChls	Bacteriochlorophylls
Bchl*a*	Bacteriochlorophylls *a*
OD_660nm_	Optical density at 660 nm
VHPR	Volumetric hydrogen production rate
SHPR	Specific hydrogen production rate
SCE	Substrate conversion efficiency
LCE	Light conversion efficiency
H_max_	Maximum cumulative hydrogen
$R_{max,H_{2}}$	Maximum rate of hydrogen production

## References

[1] Wang X., Lu Y., Chen C., Yi X., Cui H. (2024). Total-factor energy efficiency of ten major global energy-consuming countries. J. Environ. Sci..

[2] Koponen K., Braun J., Gutiérrez S.C., Evatt A., Golmen L., Guillén-Gosálbez G., Hamelin L., Jenkins S., Koljonen T., Lee C.-Y. (2024). Responsible carbon dioxide removals and the EU’s 2040 climate target. Environ. Res. Lett..

[3] Kapdan I.K., Kargi F. (2006). Bio-hydrogen production from waste materials. Enzym. Microb. Technol..

[4] Halder P., Babaie M., Salek F., Haque N., Savage R., Stevanovic S., Bodisco T.A., Zare A. (2024). Advancements in hydrogen production, storage, distribution and refuelling for a sustainable transport sector: Hydrogen fuel cell vehicles. Int. J. Hydrogen Energy.

[5] Qureshi F., Yusuf M., Kamyab H., Vo D.V.N., Chelliapan S., Joo S.W., Vasseghian Y. (2022). Latest eco-friendly avenues on hydrogen production towards a circular bioeconomy: Currents challenges, innovative insights, and future perspectives. Renew. Sustain. Energy Rev..

[6] Chandrasekhar K., Kumar S., Lee B.D., Kim S.H. (2020). Waste based hydrogen production for circular bioeconomy: Current status and future directions. Bioresour. Technol..

[7] Bartocci A., Cantelmo A., Cova P., Notarpietro A., Pisani M. (2024). Monetary and fiscal policy responses to fossil fuel price shocks. Energy Econ..

[8] Sali S., Mackey H.R. (2021). The application of purple non-sulfur bacteria for microbial mixed culture polyhydroxyalkanoates production. Rev. Environ. Sci. Bio/Technol..

[9] Corneli E., Adessi A., Dragoni F., Ragaglini G., Bonari E., De Philippis R. (2016). Agroindustrial residues and energy crops for the production of hydrogen and poly-β-hydroxybutyrate via photofermentation. Bioresour. Technol..

[10] Rai P.K., Singh S.P. (2016). Integrated dark-and photo-fermentation: Recent advances and provisions for improvement. Int. J. Hydrogen Energy.

[11] McKinlay J.B. (2014). Systems biology of photobiological hydrogen production by purple non-sulfur bacteria. Microbial Bioenergy: Hydrogen Production.

[12] Gupta S., Fernandes A., Lopes A., Grasa L., Salafranca J. (2024). Photo-Fermentative Bacteria Used for Hydrogen Production. Appl. Sci..

[13] Gómez X., Fernández C., Fierro J., Sánchez M.E., Escapa A., Morán A. (2011). Hydrogen production: Two stage processes for waste degradation. Bioresour. Technol..

[14] Keskin T., Abo-Hashesh M., Hallenbeck P.C. (2011). Photofermentative hydrogen production from wastes. Bioresour. Technol..

[15] Corneli E., Adessi A., Olguín E.J., Ragaglini G., García-López D.A., De Philippis R. (2017). Biotransformation of water lettuce (*Pistia stratiotes*) to biohydrogen by *Rhodopseudomonas palustris*. J. Appl. Microbiol..

[16] Brown B., Wilkins M., Saha R. (2022). *Rhodopseudomonas palustris*: A biotechnology chassis. Biotechnol. Adv..

[17] Li M., Ning P., Sun Y., Luo J., Yang J. (2022). Characteristics and application of *Rhodopseudomonas palustris* as a microbial cell factory. Front. Bioeng. Biotechnol..

[18] Oda Y., Samanta S.K., Rey F.E., Wu L., Liu X., Yan T., Zhou J., Harwood C.S. (2005). Functional genomic analysis of three nitrogenase isozymes in the photosynthetic bacterium *Rhodopseudomonas palustris*. J. Bacteriol..

[19] McKinlay J.B., Oda Y., Rühl M., Posto A.L., Sauer U., Harwood C.S. (2014). Non-growing *Rhodopseudomonas palustris* increases the hydrogen gas yield from acetate by shifting from the glyoxylate shunt to the tricarboxylic acid cycle. J. Biol. Chem..

[20] Montiel-Corona V., Buitrón G. (2021). Polyhydroxyalkanoates from organic waste streams using purple non-sulfur bacteria. Bioresour. Technol..

[21] Monroy I., Buitrón G. (2020). Production of polyhydroxybutyrate by pure and mixed cultures of purple non-sulfur bacteria: A review. J. Biotechnol..

[22] Thorning T.K., Raben A., Tholstrup T., Soedamah-Muthu S.S., Givens I., Astrup A. (2016). Milk and dairy products: Good or bad for human health? An assessment of the totality of scientific evidence. Food Nutr. Res..

[23] Miglietta P.P., De Leo F., Coluccia B., Vecchio Y., Capitanio F. (2021). Evaluation of virtual water and water sustainability of dairy production in Trentino Alto Adige (North-eastern Italy). Animals.

[24] Pires A.F., Marnotes N.G., Rubio O.D., Garcia A.C., Pereira C.D. (2021). Dairy by-products: A review on the valorization of whey and second cheese whey. Foods.

[25] Demirel B., Yenigun O., Onay T.T. (2005). Anaerobic treatment of dairy wastewaters: A review. Process Biochem..

[26] Adessi A., Venturi M., Candeliere F., Galli V., Granchi L., De Philippis R. (2018). Bread wastes to energy: Sequential lactic and photo-fermentation for hydrogen production. Int. J. Hydrogen Energy.

[27] Yang X., Hong J., Wang L., Cai C., Mo H., Wang J., Fang X., Liao Z. (2024). Effect of Lactic Acid Bacteria Fermentation on Plant-Based Products. Fermentation.

[28] Adessi A., De Philippis R. (2014). Photobioreactor design and illumination systems for H_2_ production with anoxygenic photosynthetic bacteria: A review. Int. J. Hydrogen Energy.

[29] Craven J., Sultan M.A., Sarma R., Wilson S., Meeks N., Kim D.Y., Hastings J.T., Bhattacharyya D. (2019). *Rhodopseudomonas palustris*-based conversion of organic acids to hydrogen using plasmonic nanoparticles and near-infrared light. RSC Adv..

[30] Liao Q., Wang Y.J., Wang Y.Z., Zhu X., Tian X., Li J. (2010). Formation and hydrogen production of photosynthetic bacterial biofilm under various illumination conditions. Bioresour. Technol..

[31] Uyar B., Eroglu I., Yücel M., Gündüz U., Türker L. (2007). Effect of light intensity, wavelength and illumination protocol on hydrogen production in photobioreactors. Int. J. Hydrogen Energy.

[32] Galli V., Venturi M., Mari E., Guerrini S., Granchi L. (2022). Selection of yeast and lactic acid bacteria strains, isolated from spontaneous raw milk fermentation, for the production of a potential probiotic fermented milk. Fermentation.

[33] Adessi A., Spini G., Presta L., Mengoni A., Viti C., Giovannetti L., Fani R., De Philippis R. (2016). Draft genome sequence and overview of the purple non-sulfur bacterium *Rhodopseudomonas palustris* 42OL. Stand. Genom. Sci..

[34] Bianchi L., Mannelli F., Viti C., Adessi A., De Philippis R. (2010). Hydrogen-producing purple non-sulfur bacteria isolated from the trophic lake Averno (Naples, Italy). Int. J. Hydrogen Energy.

[35] Touloupakis E., Chatziathanasiou A., Ghanotakis D.F., Carlozzi P., Pecorini I. (2022). Hydrogen production by immobilized *Rhodopseudomonas* sp. cells in calcium alginate beads. Energies.

[36] Carlozzi P., Sacchi A. (2001). Biomass production and studies on *Rhodopseudomonas palustris* grown in an outdoor, temperature controlled, underwater tubular photobioreactor. J. Biotechnol..

[37] Rice E.W., Bridgewater L., American Public Health Association (2012). Standard Methods for the Examination of Water and Wastewater.

[38] Adessi A., McKinlay J.B., Harwood C.S., De Philippis R. (2012). A *Rhodopseudomonas palustris nif*A* mutant produces H_2_ from NH_4_^+^-containing vegetable wastes. Int. J. Hydrogen Energy.

[39] De Philippis R., Sili C., Vincenzini M. (1992). Glycogen and poly-β-hydroxybutyrate synthesis in *Spirulina maxima*. Microbiology.

[40] Kim E.J., Lee M.K., Kim M.S., Lee J.K. (2008). Molecular hydrogen production by nitrogenase of *Rhodobacter sphaeroides* and by Fe-only hydrogenase of *Rhodospirillum rubrum*. Int. J. Hydrogen Energy.

[41] Heiniger E.K., Oda Y., Samanta S.K., Harwood C.S. (2012). How posttranslational modification of nitrogenase is circumvented in *Rhodopseudomonas palustris* strains that produce hydrogen gas constitutively. Appl. Environ. Microbiol..

[42] Dipasquale L., Adessi A., d’Ippolito G., Rossi F., Fontana A., De Philippis R. (2015). Introducing capnophilic lactic fermentation in a combined dark-photo fermentation process: A route to unparalleled H_2_ yields. Appl. Microbiol. Biotechnol..

[43] Komesu A., de Oliveira J.A.R., da Silva Martins L.H., Maciel M.W., Maciel Filho R. (2017). Lactic acid production to purification: A review. BioResources.

[44] Adamberg K., Kask S., Laht T.M., Paalme T. (2003). The effect of temperature and pH on the growth of lactic acid bacteria: A pH-auxostat study. Int. J. Food Microbiol..

[45] Hofvendahl K., Hahn-Hägerdal B. (2000). Factors affecting the fermentative lactic acid production from renewable resources. Enzym. Microb. Tech..

[46] Moradi S., Zeraatpisheh F., Tabatabaee-Yazdi F. (2023). Investigation of lactic acid production in optimized dairy wastewater culture medium. Biomass Convers. Biorefinery.

[47] Willows R.D., Kriegel A.M., Hunter C.N., Daldal F., Thurnauer M.C., Beatty J.T. (2009). Biosynthesis of Bacteriochlorophylls in Purple Bacteria. The Purple Phototrophic Bacteria.

[48] Ghosh S., Dairkee U.K., Chowdhury R., Bhattacharya P. (2017). Hydrogen from food processing wastes via photofermentation using purple non-sulfur bacteria (PNSB): A review. Energy Convers. Manag..

[49] Wang J., Wang X., Chen J., Fei Z. (2019). Experimental exploration for measurement of ammonia nitrogen in water by Nessler’s reagent colorimetry. Civ. Environ. Res..

[50] Seifert K., Waligorska M., Laniecki M. (2010). Hydrogen generation in photobiological process from dairy wastewater. Int. J. Hydrogen Energy.

[51] Wu S.C., Liou S.Z., Lee C.M. (2012). Correlation between bio-hydrogen production and polyhydroxybutyrate (PHB) synthesis by *Rhodopseudomonas palustris* WP3-5. Bioresour. Technol..

